# Facilities, challenges, attitudes, and preferences of nursing students related to e-learning in the Covid-19 pandemic in Iranian context: a cross-sectional study

**DOI:** 10.1186/s12909-024-05029-6

**Published:** 2024-01-10

**Authors:** Amirmohammad Atashinsadaf, Farhad Ramezani-badr, Tracey Long, Mohammad Imanipour, Kourosh Amini

**Affiliations:** 1 Department of Medical-Surgical Nursing, School of Nursing and Midwifery, Zanjan University of Medical Sciences, Zanjan, Iran; 2 Department of Critical Care Nursing, School of Nursing and Midwifery, Zanjan University of Medical Sciences, Zanjan, Iran; 3 Chamberlain University, School of Nursing, South Jordan, Utah USA; 4 Department of Medical Surgical Nursing, Faculty of Nursing, Aja University of Medical Sciences, Tehran, Iran; 5 Department of Psychiatric Nursing, School of Nursing and Midwifery, Zanjan University of Medical Sciences, Mahdavi St., Zanjan, Iran

**Keywords:** Covid-19, Nursing students, Online classes, e-learning, Learning style, Educational challenges

## Abstract

**Background:**

During the Covid-19 pandemic, nursing schools worldwide were forced to deliver nursing courses in ways other than the traditional face-to-face classroom setting. Numerous lessons were learned by nurse educators regarding the use of electronic and online learning strategies. It is necessary to be aware of the factors affecting e-learning and identify the strengths and weaknesses to improve the student learning experience and process in nursing school.

**Aim of study:**

The present study aimed to identify the abilities, attitudes, challenges, and preferences of nursing students regarding e-learning during the Covid-19 pandemic. Recognition of these factors can help nurse educators make needed modifications to teach more effectively.

**Methods:**

In this cross-sectional study, 228 undergraduate nursing students participated. The random sampling method used a standard questionnaire that students completed voluntarily. SPSS version 22 was used for data analysis.

**Results:**

During the Covid-19 pandemic, the majority of nursing students of Zanjan University of Medical Sciences participated in electronic classes at home using mobile phones. Students reported that logistical problems increased by participating in e-classes by their phones due to difficulty typing and frequent internet outages. Online classroom management for instructors was difficult due to students spontaneously leaving the virtual online classroom rather than attending the entire learning session. Despite the technical challenges, the attitudes of students towards the e-learning format were positive. There was a noted correlation between student attitude by gender and educational background. Students preferred that professors used more PowerPoint, showed instructional videos, and had interactive group discussion sessions rather than lecture only. Students objected to attending more than two online classes in one day if the duration lasted more than 60 min and had a rest time of fewer than 30 min between classes.

**Conclusion:**

Despite the overall positive student satisfaction with e-learning, this method of education is still far from student preferences and requires planning for an effective learning experience that meets their priorities and preferences. Using a hybrid of face-to-face and e-learning approaches together can be a more effective teaching method than either strategy alone.

## Introduction

The Covid-19 pandemic unleashed a wave of far-reaching consequences, impacting economies [1, 2], societies [1, 3], cultures [4], and public health [1, 5] on a global scale. The entire traditional educational realm, from elementary schools to institutions of higher learning, was not immune to its effects, compelling a seismic shift away from traditional in-person teaching methods. With mandatory quarantines and the suspension of in-person classes as a precautionary measure to curb the virus’s spread, educational institutions worldwide faced an unprecedented challenge [6, 7]. Faced with the uncertainty surrounding the pandemic’s resolution and the persistent need for social distancing, many educational centers and universities turned to e-learning as a means to safeguard students’ academic progress and mitigate the disruption to the education system [8, 9]. UNESCO also endorsed e-learning as a response to the closure of educational institutions [10].

E-learning (electronic learning), defined as the utilization of the internet and online technologies for educational purposes was not a novel concept before the pandemic [11]. However, the crisis accelerated its adoption in an unprecedented manner, fundamentally reshaping the landscape of information dissemination and knowledge acquisition [11]. E-learning introduced newfound flexibility and options for both educators and students, liberating education from the confines of real-time, in-person instruction in a physical classroom. Classes could be conducted asynchronously, tailored to the schedules and needs of both instructors and learners, with a resolute focus on the learner [12]. The pandemic gave rise to hybrid online courses, permitting students to progress through course material at their own pace while also engaging in scheduled live sessions with instructors. Even how examinations were taken adapted and modified to the online environment, with or without proctoring.

This surge in e-learning, especially prevalent in higher education is projected to be the future of education, with estimates suggesting that 30% of global education will transition to virtual platforms, experiencing a fifteen-fold growth in the next two years [13–15]. Given its rapid expansion, a comprehensive examination of the various factors influencing the effectiveness of online teaching and learning has become imperative [14].

Nursing education was no exception to this transformation during the Covid-19 pandemic. Nursing students, like their peers in other fields, transitioned from traditional classrooms to e-learning platforms. However, this shift was not without its unique set of challenges. Due to the extraordinary circumstances of the pandemic, the challenges brought about by this shift to online nursing education remained uncharted territory [16]. Unlike previously planned online courses, those which were developed in response to the crisis differed significantly, especially in terms of instructor and student preparedness [16]. Prior experiences with online courses in nursing schools had provided instructors ample time for curriculum development and planning of learning activities. However, the sudden imposition of mandatory quarantine measures compelled nursing faculty to rapidly adapt their teaching plans and clinical experiences to only the online format. Developing countries, such as Iran, faced even greater challenges, exacerbated by limited online infrastructure and training, which posed additional hurdles for both educators and students compared to their counterparts in developed nations [10].

To enhance the e-learning experience and ensure the education of nursing students remains uncompromised amidst the unpredictability of continued university closures due to evolving virus strains, it is vital to draw insights from past e-learning experiences and identify strengths and weaknesses [17]. In the realm of medical sciences, particularly nursing, the importance of this issue is magnified, given the direct impact of education on the delivery of quality healthcare services, a demand further intensified during the Covid-19 pandemic [18]. At a time when the need for nurses is greater than ever, the ability to produce well-prepared graduates becomes paramount.

### Review of the literature

#### Attitudes and perceptions of nursing students towards E-Learning

Previous studies have unveiled mixed attitudes and perceptions of nursing students towards e-learning. While some students perceived e-learning as a convenient and flexible alternative to traditional classroom learning others expressed a preference for face-to-face interaction, highlighting the perceived absence of social and emotional support in e-learning environments [19–24, 25, 26]. The variety of student preferences towards e-learning varies among the age and life demands of each student.

#### Challenges and barriers to E-Learning in nursing education

The literature identified numerous challenges and barriers to e-learning in nursing education. These encompassed inadequate e-learning infrastructure and resources, lack of technical support and training, limited access to reliable internet and technology, and cultural and social factors that influenced the acceptance and use of e-learning [9, 27].

#### Strategies to enhance the effectiveness of E-Learning in nursing education

The quest to enhance the effectiveness of e-learning led to the proposal of several strategies in the literature. These included providing adequate e-learning infrastructure and resources, offering technical support and training for students and faculty, addressing cultural and social factors affecting e-learning acceptance, incorporating interactive and multimedia elements into e-learning materials, and fostering social and emotional support for students through online communication and collaboration [13].

#### Relevant Studies conducted in Iran or similar contexts

Within the Iran, several studies delved into the attitudes of students towards e-learning. While some revealed positive attitudes, others illuminated a negative perception of e-learning [21–26]. Additionally, a previous study explored the technical problems induced by e-learning during the Covid-19 pandemic among instructors at Yazd University of Medical Sciences in Iran, revealing a notable increase in these problems [28].

#### Personal characteristics and demographics

It is evident from the literature that personal characteristics and demographics play a pivotal role in shaping the e-learning experience. Individuals interpret learning environments and situations differently, influenced by their personal preferences and lifestyle [29].

#### Technological Competency

Davies et al. identified access to the requisite technologies and competence in their use as critical determinants of the success of e-learning [30]. Lai’s research underscored the significance of internet competency in determining e-learning outcomes [31]. Kintu et al. revealed that a significant proportion of learners lacked the skills to effectively navigate technology-based learning components due to insufficient experience and skills in computer and internet applications, often resulting in e-learning course failures [32].

#### Physical and psychological impact

Studies have confirmed the emergence of physical and psychological problems during prolonged periods of virtual training, diminishing the effectiveness of online classes. These issues include physical pain and fatigue, vision problems, and muscle and joint pain from long periods of sitting [33].

#### The Learner’s attitude

The learner’s attitude towards e-learning emerged as a pivotal factor in its success [13, 34–36]. An e-learning program’s efficacy is contingent on the alignment of instructional design with students’ perspectives, expectations, and preferences [37, 38].

#### Unique Context of Iran

Notably, within Iran, no prior studies have examined the influence of various e-learning factors on nursing students [36]. This study marks a pioneering endeavor in Iran, exploring several facets of the e-learning process in nursing education. The results hold the promise of guiding accurate and constructive educational planning, meeting the diverse needs of contemporary nursing students, and aligning educational policy with the ultimate goal of fostering meaningful learning experiences. These efforts aim to prepare safe and competent new graduate nurses adept at harnessing the power of online e-learning methodologies.

Despite the surge in e-learning, the unique confluence of factors influencing its efficacy in nursing education during the Covid-19 pandemic remains an underexplored area of research. To meet the multifaceted needs of today’s nursing students and ensure the delivery of meaningful learning experiences, there is an imperative to delve deeper into these uncharted territories [29]. E-learning expanded by force during the pandemic, however as an educational format, it evolved and improved in a relatively short period of time and is surmised to continue as a continual purposeful educational learning tool.

#### The research questions

This study is poised to address a comprehensive array of research questions, each contributing to a more nuanced understanding of the e-learning landscape during the Covid-19 pandemic in Iran.

1. What are the technical and physical problems induced by the prolonged use of e-learning?
2. How do students perceive e-learning?
3. What preferences do students have towards e-learning?
4. Is there a correlation between students’ demographic characteristics and their attitudes and preferences towards e-learning?
5. Do the challenges associated with the long-term use of electronic tools influence students’ preferences?

In summary, this research endeavors to illuminate the complex dynamics at play in the realm of e-learning during the Covid-19 pandemic in the context of nursing education in Iran. Through an exploration of challenges, attitudes, preferences, and demographic influences, this study aspires to equip educators and policymakers with valuable insights to enhance the effectiveness of e-learning interventions in nursing education.

## Methods

### Design

This study was a cross-sectional study conducted from 10/11/2021–13/1/2022 in one Iran nursing school.

### Participants

This study was performed on undergraduate nursing students at Zanjan University of Medical Sciences, Iran. The criteria for admission to the study were: (1) Passing at least one semester of an e-learning course, (2) Participant consent to participate in the study, and (3) Absence of any known chronic physical or psychological problems as reported by the student, that may significantly impact the student’s ability to engage in e-learning activities. The purpose of removing a student from the study who has chronic physical or psychological problems was to remove any factors that may interfere with the data collection of possible complications from e-learning such as headaches and vision disturbances, which were assessed during the study. The number of eligible students to enter the study was 513 students.

### Sampling

We used Cochran’s formula to determine the sample size: (p = q = 0.05, Z = 1.96, Power = 0.8, α = 0.05, *N* = 513, *n* = 220). The main variables included facilities, attitudes, preferences, and challenges. We added 10% to the calculated sample size for incomplete questionnaires. Therefore, 242 students were entered into the study by simple random sampling using a random number table. Out of 242 distributed questionnaires, fourteen students did not answer the questionnaire questions completely and were eliminated from the study. Two hundred twenty-eight questionnaires were analyzed for the study. The sample was a simple convenience sample as all participants came from the same university.

### Data collection

Upon obtaining approval from the Institutional Review Board (IRB), a comprehensive explanation of the study was provided to the participants. Written consent was obtained from each selected student prior to their inclusion in the study. There was no cost associated with participating in the study, and participants were not provided any form of monetary compensation. Furthermore, no anticipated negative complications were expected as a result of their involvement in the study.

One of the objectives of this study was to investigate the physical and mental effects of e-learning. It is important to note that these effects were not caused by the study itself, as it aimed to conduct a phenomenological examination of students who were already engaged in e-learning due to the widespread adoption of this educational approach resulting from the Covid-19 pandemic.

### Ethical considerations

Biomedical Research Ethics Committee approved the proposal of the present study with the code: IR.ZUMS.REC.1400.295. Students were not coerced to participate in the study, nor were any grades affected by their participation. The anonymity of the students was protected for participating in the research. Permission from the developer of the research tool was obtained via email.

### Instruments

We used two questionnaires to collect the data. The first questionnaire was designed to gather information about the demographic and educational characteristics of the students. This questionnaire included questions about age, gender, marital status, school of study, grade of education in the university, total grade point average, place of residence, and economic level of income. The questionnaire was specifically designed for the students.

The second questionnaire was developed by Singh et al. (2021) in India and was used in a previous study [6]. The questionnaire consisted of 33 questions and six sections. In the first part of the questionnaire, five questions were asked about the devices needed to participate in e-learning and the student’s ability to use these tools. In the second part, ten questions examined common teaching methods by the professors. In the third part, three questions examined physical and psychological problems before and during electronic learning. In the fourth part, a question with nine items on a five-point Likert scale assessed students’ attitudes toward e-learning compared to face-to-face teaching methods. The fifth part included five questions to determine student preferences regarding e-learning. At the end of the questionnaire, one open question (question No 33) allowed the student to mention any issues or experiences with e-learning in a way that was not mentioned in the other questions.

In this article, due to the extensive volume of data collected and the unique nature of each dimension in the questionnaire, we have chosen to focus on presenting four out of the six study dimensions, with a specific emphasis on e-learning as a separate entity.

As the Singh and colleagues’ questionnaire was not initially intended for Iranian populations, we conducted a translation process to convert it into Persian. This involved utilizing a forward-backward translation method. Following the translation, we sought input from 15 nursing and medical education experts who were professors and faculty members. We gathered their feedback, which was carefully considered for making necessary modifications to the questionnaire. To ensure the questionnaire’s content validity, we conducted two sessions with the entire research team, incorporating the suggestions and feedback received from the experts. Adjustments were made to enhance its suitability for the target population.

To assess the Content Validity Index (CVI), the revised questionnaire was distributed to 15 faculty members and nursing students. The total CVI was calculated using the Lawshe Table [39], resulting in a value of 0.98, which is considered acceptable. Additionally, the Content Validity Ratio (CVR) for relevance, clarity, and simplicity was determined to be 0.89, 0.93, and 0.90, respectively, indicating satisfactory values. Additionally, the exploratory factor analysis was conducted on the questionnaire to examine its underlying factor structure, which was confirmed. The analysis was performed using principal component analysis (PCA) with varimax rotation. The sample consisted of 228 students, and the Kaiser-Meyer-Olkin (KMO) measure of sampling adequacy was found to be 0.782, indicating an acceptable sample for factor analysis.

Part (1) Devices and Ability to Use Tools: Questions about e-learning devices and student proficiency formed the “Technological Competence” factor (Factor loading: 0.68–0.82). Part (2) Professors’ Teaching Methods: Questions about teaching methods yielded the “Teaching Strategies” factor (Factor loading: 0.61–0.79). Part (3) Physical-Psychological Problems and Control Methods: Questions on physical-psychological issues and control methods formed the “Well-being and Coping” factor (Factor loading: 0.72–0.86). Part (4) Attitudes Towards E-Learning vs. Face-to-Face Teaching Methods: A single question measuring attitudes toward e-learning vs. traditional teaching loaded on the “Attitudinal Preference” factor (Factor loading: 0.71–0.88). Part (5) Preferences Regarding E-Learning: The five questions exploring preferences regarding e-learning loaded significantly on a factor termed “Learning Environment” (Factor loading range: 0.64–0.79). Open-Ended Question: An open-ended question allowed students to share additional e-learning experiences or issues, however, it was not analyzed with the other factors.

To assess the reliability of the questionnaire, we used the test-retest method and calculated the Cohen’s Kappa coefficient. The test-retest analysis resulted in a correlation coefficient value of 0.79, indicating a high level of instrument reliability. Additionally, the Cohen’s Kappa coefficient was calculated to be 0.82, further confirming the questionnaire’s reliability.

### Analyses

To analyze the data, we used SPSS version 22. We used Shapiro-Wilk and Kolmogorov-Smirnov tests to determine the compliance of quantitative variables with the normal distribution, and the results showed that the data followed the normal distribution. The Pearson (r) test was used to measure the quantitative correlation between two variables. Spearman’s test (ρ) was used to measure the correlation between two ordinal variables, or the dependent variable, which was ordinal. Eta (η) test was used to measure the correlation between nominal-distance variables. V-Kramer (V) and Lambda (λ) tests were used to examine the correlation between the two variables, nominal qualitative. In this study, *p* < 0.05 was considered significant.

## Results

### Profile of participants

The participants had a mean age of 22.52 ± 2.56, and their overall grade point average (GPA) was 16.60 ± 1.27, based on the grading system used at the Iranian University. Out of the participants, the most were female (122, 53.5%), and were single (205, 89.9%). The majority of the students, resided in a small town/city (202, 88.6%). Additionally, 128 students (56.1%) had an average economic status **(**Table 1**)**.

**Table 1:** Description of the students based on demographic variables

Variables		
Gender **n (%)**	Male	106 (46.5)
	Female	122 (53.5)
Marital status **n (%)**	Single	205 (89.9)
	Married	23 (10.1)
Grade **n (%)**	1	27 (11.8)
	2	72 (31.6)
	3	69 (30.3)
	4	60 (26.3)
University entrance semester **n (%)**	First/Fall	125 (54.8)
	Second/Spring	103 (45.2)
Living place **n (%)**	Rural	26 (11.4)
	Town	137 (60.1)
	City	65 (28.5)
Economic status **n (%)**	Very weak	5 (2.2)
	Weak	10 (4.4)
	Medium	128 (56.1)
	Good	77 (33.8)
	Very good	8 (3.5)
Age (year) **Mean ± SD**	22.52 ± 2.56	
TGP Score (out of 20) **Mean ± SD**	16.60 ± 1.27	

Abbreviations: n, number of participants; SD, standard deviation; TGP, total grade point average

### The level of students’ access to the technology and the skills to use them

Results showed that the majority of students accessed e-learning from their personal home (220, 96.5%), used a personal mobile phone (192, 84.2%), had internet access for their mobile phone (187, 82.0%) without disturbance, and had access to a quiet environment for online classes (171, 75.0%). Results showed that the majority of nursing students had an average knowledge and skill level of accessing the internet (127, 55.7%) and using mobile phones (127, 55.7%) and computers (109, 47.8%). The most common platform for holding electronic classes by professors and officials was the Navid system (224, 98.2%).

### Prevalence of physical and psychological problems in online and offline classes

Among the various physical and psychological problems associated with participating in online and offline classes, general physical fatigue and eye fatigue were the most frequently reported issues at the “frequent” and “always” levels, with respective prevalences of 68 (29.8%) and 67 (29.4%). Following these, loss of focus had a prevalence of 44 (19.3%), while back and neck pain had a prevalence of 43 (18.8%). The least frequently reported issue was earache, with a prevalence of 15 (6.6%) (Table 2).

**Table 2:** Physical and psychological problems reported in long-term e-learning

Problems		n (%)
Headache	Never	73 (31.6)
	Rarely	77 (33.8)
	Sometimes	51 (22.4)
	Frequently	20 (8.8)
	Always	8 (3.5)
Earache	Never	144 (63.2)
	Rarely	45 (19.7)
	Sometimes	24 (10.5)
	Frequently	10 (4.4)
	Always	5 (2.2)
Eye fatigue	Never	43 (18.9)
	Rarely	45 (19.7)
	Sometimes	73 (32.0)
	Frequently	39 (17.1)
	Always	28 (12.3)
Fatigue	Never	38 (16.7)
	Rarely	50 (21.9)
	Sometimes	72 (31.6)
	Frequently	47 (20.6)
	Always	21 (9.2)
Anxiety	Never	86 (37.7)
	Rarely	62 (37.2)
	Sometimes	42 (18.4)
	Frequently	28 (12.3)
	Always	10 (4.4)
Loss of focus	Never	95 (41.7)
	Rarely	48 (21.1)
	Sometimes	41 (18.0)
	Frequently	32 (14.0)
	Always	12 (5.3)
Sleep disorder	Never	92 (40.4)
	Rarely	67 (29.4)
	Sometimes	38 (16.7)
	Frequently	21 (9.2)
	Always	10 (4.4)
Back and neck pain	Never	96 (42.1)
	Rarely	50 (21.9)
	Sometimes	39 (17.1)
	Frequently	30 (13.2)
	Always	13 (5.7)

Abbreviations: n, number of participants

### Student challenges related to the use of digital facilities

Of the 228 participants, more than half of the students (124, 54.4%) reported that online classes would be held for them due to Covid-19 in an effort to help them continue in their education plan towards graduation. The most common challenges faced by students in any online/offline classroom with digital devices were problems typing in Persian or English, and internet outages causing them to leave the online classroom during a live class (Table 3).

**Table 3:** Challenges related to the use of digital facilities and presence in online and offline classes

Challenges		n (%)
Differences in the synchronization of audio and video playback	Never	24 (10.5)
	Rarely	71(31.3)
	Sometimes	24 (10.5)
	Frequently	3(1.3)
	Always	2(0.9)
Disconnect the internet and exit the class	Never	17(7.5)
	Rarely	60(26.3)
	Sometimes	39(17.1)
	Frequently	7(3.1)
	Always	1(0.4)
Lack of clarity in the voice of classmates/teachers	Never	18(7.9)
	Rarely	67(29.4)
	Sometimes	33(14.5)
	Frequently	14(1.8)
	Always	2(0.9)
Problems playing videos	Never	19(8.3)
	Rarely	73(32.0)
	Sometimes	25(11.0)
	Frequently	5(2.2)
	Always	2(0.9)
Error sharing files	Never	20(8.8)
	Rarely	73(32.0)
	Sometimes	25(11.0)
	Frequently	5 (2.2)
	Always	1 (0.4)
Problems typing in Persian/English	Never	30 (13.2)
	Rarely	43 (18.9)
	Sometimes	22 (9.6)
	Frequently	13 (5.7)
	Always	16 (7.0)

Abbreviations: n, number of participants

### Students’ attitudes toward e-learning

As depicted in Table 4, 155 (68%) of nursing students agreed there was a negative impact of Covid-19 on the quality of their education. 176 (77.2%) of them stated that they did not have opportunities for face-to-face classes in the e-learning setting. Despite the challenges with technology, 120 (52.7%) believed that the e-learning method was the best option to continue their education during the Covid-19 pandemic. 168 (73.7%) agreed that the e-learning option was more available during the pandemic than before. Most participants thought continuing their learning via the online courses caused less stress and anxiety compared to the fear of contracting the virus or other communicable infections through the traditional face-to-face method. 156 (68.4%) of students considered e-learning to be economically viable. However, most students (134, 58.8%) believed that interaction with others in the electronic form is weak and lacked the ability to make personal connections with their peers and professors. Most students thought that e-learning methods could not replace face-to-face training in the future.

**Table 4:** Students’ attitudes toward e-learning

Items	Strongly agree n (%)	Agree n (%)	Neither agrees nor disagree n (%)	Disagree n (%)	Strongly disagree n (%)
The Covid-19 pandemic has hurt my education.	82 (36.0)	73 (32.0)	32 (14.0)	35 (15.4)	6 (2.6)
With virtual classes, I do not have the experience same as attending formal classes.	73 (32.0)	103 (45.2)	22 (9.6)	20 (8.8)	10 (4.4)
Virtual training is the best training method during the Covid-19 pandemic.	30 (13.2)	90 (39.5)	62 (27.1)	29 (12.7)	17 (7.5)
The possibility of repetitive reading in virtual education makes learning easier.	32 (14.0)	101 (44.3)	61 (26.8)	25 (11.0)	9 (3.9)
Virtual training is more accessible (spatial/temporal) than face-to-face training.	42 (18.4)	126 (55.3)	29 (12.7)	21 (9.2)	10 (4.4)
Virtual education reduces the anxiety and stress of getting Covid-19 disease more than face-to-face training.	54 (23.7)	119 (52.2)	39 (17.1)	14 (6.1)	2 (0.9)
Virtual education is more economical than face-to-face education.	45 (19.7)	111 (48.7)	39 (17.1)	23 (10.1)	10 (4.4)
Interaction with teachers and other classmates is more than face-to-face education in virtual classrooms.	11 (4.8)	45 (19.7)	38 (16.7)	82 (36.0)	52 (22.8)
In the future, virtual education can replace face-to-face education for students.	22 (9.6)	53 (23.2)	55 (24.1)	48 (21.1)	50 (21.9)

Abbreviations: n, number of participants

### Students’ preferences in e-learning

As displayed in Table 5, the three methods that students preferred for e-learning were: (1) receiving the content for lessons through PowerPoint presentation by the instructor (161, 70.6%), (2) showing educational videos (160, 70.2%), and (3) holding an interactive session alongside a group discussion throughout the course (108, 47.4%).

**Table 5:** Students’ preferences in e-learning

Preferences	n (%)
Lecture without PowerPoint and video displaying	39 (17.1)
Online whiteboard with descriptive charts	49 (21.5)
Only PowerPoint	161 (70.6)
Problem-based /case-based training	92 (40.4)
Interactive session with group discussion/role-playing	108 (47.4)
Tutorial videos	160 (70.2)
Recorded professors’ lectures	101 (44.3)
Using pamphlets and e-books	55 (24.1)
Using PDF and Word files	94 (41.2)
Question and answer	101 (44.3)
Flipped class	35 (15.4)

Abbreviations: n, number of participants

### Students’ suggestions for using e-learning

To improve the quality of e-learning, most students (191, 83.7%) preferred that the instructor upload the educational content to the online course platform before holding the class, believing that this method would help the students be more prepared to benefit from the live class. 166 (72.8%) of students also desired watching recorded lectures by professors ahead of time to promote engagement and absorption of the material before meeting with the class. Half of the students (111, 116, 50.7%) appreciated the ability to continue learning with e-learning methods even after the course was completed. Students believed each online class session should not last more than 60 min. They should not have more than two online classes in one day and should rest for at least 30 min between two online sessions (Table 6).

**Table 6:** Students’ suggestions for promoting e-learning and its times

Suggestions	Response	n (%)
Uploading content by professors a few days before	Strongly agree	55 (24.1)
	Agree	136 (59.6)
	No idea	27 (11.9)
	Disagree	8 (3.5)
	Strongly disagree	2 (0.9)
The teaching of a part of the lessons by the student	Strongly agree	25 (10.8)
	Agree	91 (39.9)
	No idea	71 (29.2)
	Disagree	32 (14.0)
	Strongly disagree	14 (6.1)
Sharing recorded lectures by professors	Strongly agree	36 (15.8)
	Agree	130 (57.0)
	No idea	51 (22.4)
	Disagree	11 (4.8)
	Strongly disagree	0 (0.0)
Duration of classes (minutes) **Mean ± SD**	62.12 ± 22.11	
Number of classes per day **Mean ± SD**	2.03 ± 1.2	
Rest time (minutes) **Mean ± SD**	34.27 ± 24.57	

Abbreviations: n, number of participants; SD, standard deviation

### The level of students’ access and use of e-learning

This study revealed a relationship between demographic variables and the level of students’ access to the facilities. The present study showed that there is a statistically significant relationship between the economic status of students and the use of more expensive digital tools, including computers/laptops (τ = 0.710, *p* = 0.020) and personal tablets (τ = 0.487, *p* = 0.043) to participate in electronic classes. A statistically significant relationship was observed between using Wi-Fi at home as an Internet source and economic status (τ = 0.327, *p* = 0.006). The present study’s findings showed that the use of mobile phone data to the Internet source had a direct relationship with the increase in a student’s GPA (η = 0.499, *p* = 0.009). Also, a statistically significant relationship was observed between having suitable environmental conditions for participating in electronic classes with each of the variables of marital status (V = 0.435, *p* = 0.041) and place of residence (V = 0.721, *p* = 0.039). There was a statistically significant relationship between computer skills and students’ economic status (ρ = 0.221, *p* = 0.024). Not surprising, and now confirmed by this data, students who had more stable living environments and a higher economic status to purchase Internet service and electronic devices had increased skills and resultant academic success compared to those who did not have these facilities.

### Relationship between demographic variables and students’ problems in e-learning

The results showed a statistically significant relationship between female gender and physical-psychological problems such as headaches and eye strain before (V = 0.316, *p* = 0.048) and after the start of electronic classes (λ = 0.267, *p* = 0.036).

### Relationship between demographic variables with students’ attitudes and preferences

The results showed a statistically significant relationship between attitudes toward e-learning with the variables of gender (η = 0.229, *p* = 0.000) and year of university entry (η = 0.020, *p* = 0.000). The present study results showed a statistically significant relationship between the preferences of students in different methods of e-learning with the year of entering the university or the students’ educational level (V = 0.291, *p* = 0.002). A statistically significant relationship was observed between the students’ preference of having the instructor upload content a few days before the class as a way to promote the quality of the e-learning with those who had a higher total grade point average (η = 0.104, *p* = 0.031) and more years studying at the university (V = 0.177, *p* = 0.046). There was a statistically significant correlation between students’ preference in active learning by having students teach a part of the course for active learning and those students with a higher total grade point average (η = 0.248, *p* = 0.006) and more years of university study (V = 0.200, *p* = 0.007). There was a statistically significant relationship between students’ preference to have access to the professor’s pre-recorded lectures with the total grade point average (η = 0.01, *p* = 0.043). There was a statistically significant relationship between students’ preferences regarding the number of electronic classes per day with the gender variable (V = 0.240, *p* = 0.042). Female students preferred less classes per day and more time in-between online classes for resting.

### Relationship between the level of students’ access to the facilities and students’ attitudes toward e-learning

The present study showed that there was a significant relationship between the attitude of students towards electronic learning and each of the variables of the tools used to participate in electronic classes (η = 0.626, *p* = 0.025), the source of the Internet (η = 0.321, *p* = 0.002) and suitable environmental conditions for participating in classes (η = 0.449, *p* = 0.042). There was a statistically significant relationship between students’ attitude towards e-learning and their ability to use computers (η = 0.300, *p* = 0.020)., phones (η = 0.881, *p* = 0.001).and the Internet (η = 0.655, *p* = 0.012).

### Relationship between the level of student access to the facilities and student preferences

There was a statistically significant relationship between students’ preference for the number of electronic classes during the day with each of the variables of skills in using mobile phones (η = 0.121, *p* = 0.0312), computers (η = 0.410, *p* = 0.040) and the Internet (η = 0.466, *p* = 0.031). There was a statistically significant relationship between the students’ preference for the time of any electronic classes during the day with each of the variables of skill in using mobile phones (η = 0.324, *p* = 0.040), computers (η = 0.401, *p* = 0.012) and the Internet (η = 0.523, *p* = 0.041).

### Relationship between challenges and students’ attitudes in e-learning

There was a significant relationship between students’ attitude towards e-learning and each of the physical problems, including headache (V = 0.360, *p* = 0.006), body fatigue (V = 0.426, *p* = 0.010), and eye fatigue (V = 0.756, *p* = 0.001). As physical problems and discomforts increased, so did their negative attitude towards e-learning increase. Also, there was a significant relationship between the negative problems encountered during participation in electronic classes such as frequent internet outages (η = 0.323, *p* = 0.025) and difficulties in typing Persian/English words (η = 0.401, *p* = 0.028) with students’ negative attitudes towards electronic learning.

### Relationship between challenges and students’ preferences in e-learning

There was a significant relationship between students’ preferred time for each e-class and the difficulty in sharing files by the student (η = 0.302, * p* = 0.022). There was a statistically significant relationship between the number of students who preferred electronic classes in a day and the problem of lack of clarity in the voice of classmates and teachers (η = 0.289, *P* = 0.034). There was a statistically significant relationship between the physical-psychological problems created after the start of e-classes with student preference to promote active learning by uploading educational materials from a few days before the holding of e-classes by professors (V = 0.320, *p* = 0.000).

## Discussion

This study aimed to determine the abilities, attitudes, challenges, and preferences of nursing students in relation to e-learning during the Covid-19 pandemic. The results of the present study showed that the majority of students accessed online courses from their homes (96.49%), used personal mobile phones (84.2%), used the Internet without disturbance (82.0%), and participated in electronic classes in a quiet environment to optimize online learning (75.0%). In the research of Singh et al., conducted in 2021 among nursing and medical students in India [6], mobile phones and using internet data have been the most used. Data showed that functional and basic skills and knowledge of using electronic tools of most students were at an average level (47.8%). Kamalian’s study (2009) also mentioned the average technology skill level of students requires the attention of educational designers in the learning environment to create training to improve this skill in students [40]. During the Covid pandemic, the skill of using mobile phones (55.7%) and the Internet (53.5%) has been at an average level.

The results showed that the prevalence of physical problems, such as eye problems, ear problems, sleep disorders, headaches, neck pain, back problems, psychiatric problems, such as anxiety, and impaired concentration after attending e-classes was higher than before using online learning. Among these, body fatigue, eye fatigue, and headache were the most common problems after attending online classes, which were not unexpected. Spending long hours with digital tools for multiple courses during the day and several semesters without prior planning could be associated with such problems due to the sudden change in the learning environment during the pandemic and quarantine. Consistent with our study, in India, Agarwal et al. (2021) showed that the requirement to use electronic teaching methods during the Covid-19 pandemic had adverse effects on physical and mental health [41]. The study of Singh et al. in 2021 showed that the incidence of physical and mental problems related to virtual classes among medical and nursing students in India had also increased significantly [6].

This current study showed that after starting and participating in e-classes, more students used pharmacological and non-pharmacological approaches to control or reduce physical-psychological problems than before electronic learning methods were used. In the study by Singh et al., almost one-third of Indian students who complained of headaches, neck pain, and back pain used over the counter or prescribed painkillers during the Covid electronic learning era [6].

This study revealed that the most significant challenge in online classes for students was difficulty typing Persian into English, followed by the internet outage and spontaneously losing Internet connectivity to the online course. A study conducted at Marvdasht University in 2021 by Yarizanganeh et al. also showed that the high incidence of Internet outages caused lack of connectivity for students and was the main problem for Iranian students using online learning [42].

The present study showed that most students considered the Covid-19 pandemic negatively impacted their education. They believed that attending e-classes during the Covid-19 pandemic and the mandatory quarantine period did not give them the needed learning experiences compared to the benefits of attending a face-to-face class. However, most students considered the electronic method the best teaching method available during the Covid-19 pandemic. Students believed that the e-learning method is more economical and more accessible to facilitate learning due to the constant availability of online classroom content. An advantage that most students experienced by using the electronic method was the reduction of anxiety and stress in contracting infectious diseases, including Covid-19. However, they did not consider the quality and quantity of communication and interaction with teachers or classmates satisfactory. 43.06% of the participants were against only using virtual teaching methods to replace traditional face-to-face teaching methods in the future, and 33.8% of students believed that although the electronic method could continue to be used, the face-to-face teaching method should be used in the future.

Overall, students had a positive attitude toward e-learning as it allowed them to continue making progress in their plan of study towards graduation. This finding was consistent with some studies conducted in the past [19, 20, 22–24]. Other studies have shown opposite results for student preferences in which students were only interested in face-to-face education and would wait until after the pandemic to have the live option of traditional classroom learning [25, 26]. Given the conflicting findings, it is recommended to provide training in a hybrid combination of electronic and face-to-face methods.

The present study showed that most nursing students want their education to include the use of succinct PowerPoint lectures, instructive videos, interactive sessions with group discussions, and role-playing. Preferred teaching methods that involve both lecture and visual presentations have commonly been requested by students, suggesting professors should use a variety of active learning strategies to meet the needs of today’s higher education students.

According to a study by Singh et al. in 2021, a large number of students preferred the professor upload educational materials a few days before the online classes, in addition to actively teaching part of the course by the students themselves and sharing the lectures with the professors to improve the quality of the online learning. A significant percentage of nursing students agreed with these methods promote active learning [6]. According to the nursing students in the present study, electronic classes with an average duration of 60 min and two classes per day with an average rest time of 30 min between classes were considered the most effective. In the study by Singh et al. (2021), Indian students preferred to have 3 to 6 lessons, each lasting less than 40 min, with a break of at least 10 to 20 min between classes [6].

The present study showed a statistically significant relationship between access to digital tools to participate in electronic classes and economic status. Consistent with Singh et al.‘s study, the access to use personal laptops/computers and tablets to participate in classes was higher among students with good and very good economic status [6]. The study found the most common tool among students to access the Internet is a mobile phone. Considering the average economic status of the majority of students to afford a mobile phone rather than a personal computer, it is recommended that professors should design educational materials to display better according to a phone screen layout such as vertical images rather than large spreadsheets or horizontal content.

A statistically significant relationship was observed between the variable of access to the Internet and the variables of student total grade point average and economic status. This finding was also observed in Singh et al.‘s study [6]. The use of home Wi-Fi as an Internet source was more common in students with good and very good economic status. However, using mobile phone data rather than free Wifi to access the Internet directly correlated with the increase in the grade point average. In explaining the possible cause of this finding, it may be due to higher income of students who could afford personal data and the lack of location restriction compared to other internet resources used by students, as well as the student’s greater skill in working with mobile phones.

A statistically significant relationship was observed between the variables of suitable environmental conditions for participation in electronic classes and marital status. In can be surmised that married people had more suitable environmental conditions to participate in classes, due to living in a house independent from the father’s house, with less people accessing the Internet. Also, there were less favorable environmental conditions for students living remotely in the center of the province than those in the city. The conditions were more plentiful for the students living in the city than the students living in the village, as the study of Singh et al. also discovered [6], Students living in rural environments often had more work responsibilities compared to those in the city and those of lower economic status often lived in more rural areas. Large families could be obstacles to having suitable and undisturbed environmental conditions to participate in electronic classes for students living in villages.

Similar to the findings of Singh’s study, this study found there was a statistically significant relationship between computer network literacy and the economic status of students. The computer literacy in students with a good financial situation was higher than students with an average economic level who rated as weak and very weak [6].

This study also showed a statistical relationship between physical-psychological problems before and after the start of electronic classes and the gender variable. These problems were reported more often in the female student population than in male students, which should be considered in future studies. In general, the prevalence of all kinds of disorders was higher in women after participating in the online classes. Among these, body fatigue, eye fatigue, and headache were the most prevalent in both genders. According to the study of Singh et al. [6], the occurrence of these disorders had a direct and significant relationship with the amount of each electronic class, the number of classes during the day, and the underlying diseases of the students.

Contrary to some studies conducted in the past there was a significant difference between attitudes toward virtual classes by gender [22, 23, 43]. In this study women had a more positive attitude than men about e-learning. The findings regarding preference of e-learning by gender in the present study are consistent with several other studies [21, 44–46]. Furthermore, the study revealed a significant correlation between the year of enrollment in university and the attitude towards e-learning. Surprisingly, students with lower grades exhibited a more favorable attitude towards e-learning compared to students with higher grades. However, this finding is in contrast to some previous studies that have suggested that students’ academic performance and educational level are not related to their attitudes towards e-learning [21, 45–47].

Students of the upper semesters were most interested in audio-visual learning methods. There was only a significant relationship between gender and students’ preferences for the number of electronic classes per day among the demographic variables. Women preferred to hold a maximum of two classes per day, and men chose to have more classes during the day.

Uploading the class content before the live online class was the preference of senior students. The students of the lower semesters preferred teaching a part of the course with their peers. They believed it was a way to improve the quality of active learning in the e-learning method. According to this finding, paying attention to arranging course plans for upper and lower-semester students is important. The three preferred methods of uploading educational materials, teaching a part of the course by the student, and sharing the professors’ lectures with students were directly related to the student’s higher grade point average. This finding also can guide professors in how they should organize and plan for online classes.

The findings indicated a significant relationship between students’ attitudes towards electronic learning with each of the variables of the tools used to participate in electronic classes, the Internet source, and suitable environmental conditions for participating in classes. More students who used computers or tablets had a good economic situation and had a separate room away from crowds and disturbance to participate in electronic classes. This is consistent with the findings of Habibi et al. in 2015 [48]. There is a statistically significant relationship between students’ attitude towards e-learning and their ability to use computers, mobile phones, and the Internet, such that students with high computer and technology skills had a positive attitude towards e-learning compared to students with low skills. They had little use of these tools which is consistent with the studies of Ojaghi et al. (2019) [49], Gorghiu et al. (2010) [50], Seyede Naqhavi (2007) [51] and Shiri et al. (2012) [52].

There was a significant relationship between the skill of working with mobile phones, computers, and the Internet with the preferred number and time of e-class per day. Students with more knowledge and skills in using mobile phones, computers, and the Internet agreed to hold more electronic classes each class during the day compared to those with less skill who preferred fewer e-learning classes daily.

There was a significant relationship between students’ attitudes toward e-learning and physical problems, including headache, body fatigue, and eye fatigue. Most students who suffered from any of the above physical problems after participating in electronic classes had a negative attitude towards participating in these classes. Also, there was a significant relationship between students’ attitudes towards e-learning and digital problems created during online classes, so students who had frequent internet outages and problems in Persian/English typing expressed a negative attitude in the e-learning experience.

The present study showed a significant relationship between the problems of file-sharing and the preferred length of time for each e-class. As file sharing and file retrieval problems increased, students preferred more time to upload educational materials and assignments posted to the virtual education system. Students preferred to reduce the number of electronic classes per day due to an increase in the problem of lack of understanding the digital or written voice of classmates and teachers, which could be due to fatigue from continuing to attend online classes.

Most students with underlying health conditions wanted to receive instructional materials and pre-recorded lectures from professors several days ahead of the online class to promote their e-learning.

There was a relationship between the physical-psychological problems created after the e-learning classes with students’ preference of uploading educational materials a few days before the online class. During the several days between the professor uploading the materials and the beginning of the online class students preferred to read the uploaded educational materials in small volumes many times to reduce the physical and mental load on the student. Students who had sleep disorders after attending e-classes also had the highest preference with this preparatory method.

### Limitations

One limitation of the present study was the limited literature review and lack of previously published studies on the physical and psychological problems created during e-learning and student preferences regarding e-learning. Another significant limitation of this study was the impossibility of generalizing the results to other communities due to the impact of the social, economic, and cultural factors on the variables that were studied with nursing students from Iran. This study was limited to Iran nursing students with sometimes limited access and Internet support to effectively complete online courses and may not be generalizable to other communities or countries.

## Conclusions

During the Covid-19 pandemic at Zanjan University of Medical Sciences, this study delved into the intricacies of nursing students’ engagement in electronic classes, revealing key insights into their abilities, attitudes, challenges, and preferences regarding e-learning. The findings underscored the prevalent trend of Iranian nursing students accessing online courses from the comfort of their homes, predominantly using personal mobile phones. Comparable studies in India emphasized the widespread use of mobile phones and internet data among medical and nursing students, indicating a parallel technological landscape.

Despite the convenience of electronic classes, a notable escalation in physical and psychosocial problems among nursing students emerged, encompassing issues like eye fatigue, headaches, and anxiety. The extended hours spent with digital tools, exacerbated by the abrupt shift to online learning during the pandemic, were identified as potential catalysts for these physical challenges. The adoption of pharmacological and non-pharmacological approaches to mitigate these issues post-e-learning reflected students’ resilience and adaptive strategies.

Additional challenges surfaced, including difficulties in typing Persian into English, internet outages and sporadic loss of connectivity, which created hurdles to the seamless flow of online courses. Students expressed mixed sentiments about the impact of the Covid-19 pandemic on their education, acknowledging the economic and accessibility advantages of e-learning but lamenting the challenges of the online experience compared to traditional face-to-face classes.

Students advocated for a hybrid approach, blending electronic and face-to-face methods, addressing the conflicting preferences revealed in the study. The ideal teaching methods, as voiced by nursing students, included succinct PowerPoint lectures, instructive videos, interactive group discussions, and role-playing sessions. Furthermore, they expressed their preferred class duration, number of classes per day, and breaks between sessions as crucial for effective e-learning.

Economic disparities were evident in students’ access to digital tools, highlighting the need for accommodating mobile-centric educational materials for those with limited access to personal computers. Additionally, the study discerned correlations between Internet access, academic performance, and suitable environmental conditions, emphasizing the intricate interplay of socio-economic factors in the e-learning landscape.

While acknowledging the overall satisfaction with e-learning during the pandemic, it is evident that the current educational paradigm falls short of aligning with all student preferences. This underscores the imperative for nursing educators and curriculum developers to address these gaps to deliver effective education to cultivate competent and resilient nurses.

### Recommendations

Considering the study’s findings, several recommendations are proposed to enhance the efficacy of online education for nursing students:

#### Mitigating physical and psychosocial issues

Curriculum developers should proactively address the physical and psychosocial challenges associated with e-learning, tailoring solutions to the unique needs of nursing learners. Recognizing the potential for eye fatigue, instructors of online courses should customize physical breaks for the learners.

#### Adapting to Student Preferences

To optimize virtual education, consideration should be given to students’ preferences regarding class duration, frequency, and inter-session breaks. *Improving Internet Infrastructure*: Communication and internet service providers are urged to enhance internet platforms, ensuring strong broad bandwidth and seamless connectivity as possible.

#### Comprehensive E-Learning Management

Future studies should encompass a broader spectrum of factors influencing e-learning to formulate a more comprehensive strategy for educators to manage online education effectively.

#### Cross-cultural studies

To gain a deeper understanding of the factors influencing e-learning, additional studies should explore diverse societies and cultures, providing a more global perspective.

#### Computer Literacy Education

Nursing students should receive comprehensive training in computer literacy and internet access skills to empower them for active participation in e-learning and online courses.

## Data Availability

The datasets used and/or analyzed during the current study are available from the corresponding author on request.
